# Efficacy and safety of sacubitril/valsartan in patients on peritoneal dialysis: a systematic review and meta-analysis

**DOI:** 10.1590/2175-8239-JBN-2025-0318en

**Published:** 2026-03-30

**Authors:** Caio Lima da Silva, Pandora Eloa Oliveira Fonseca, Viviane Calice-Silva, Daniela Ponce, Paulo Novis Rocha, Maria Gabriela Guimarães

**Affiliations:** 1 Universidade Federal do Rio Grande do Norte, Natal, RN, Brazil.; 2 Universidade Federal de Campina Grande, Campina Grande, PA, Brazil.; 3 Fundação Pró-Rim, Joinville, SC, Brazil.; 4 Universidade de Região de Joinville, Joinville, SC, Brazil.; 5 Universidade do Estado de São Paulo, Departamento de Medicina Clínica, São Paulo, SP, Brazil.; 6 Universidade Federal da Bahia, Departamento de Medicina Clínica, Salvador, BA, Brazil.; 7 Universidade Federal da Bahia, Programa de Pós-Graduação em Ciências da Saúde, Salvador, BA, Brazil.; 8 International Renal Research Institute of Vicenza, Vicenza, Italy.

**Keywords:** Peritoneal Dialysis, Sacubitril-Valsartan, Kidney Failure, Chronic, Heart Failure, Cardio-Renal Syndrome

## Abstract

**Background:**

Sacubitril/valsartan is a recommended medication for managing heart failure (HF). However, its role in peritoneal dialysis (PD) patients remains uncertain. We conducted this systematic review and singlearm meta-analysis to assess the efficacy and safety of sacubitril/valsartan in this population.

**Methods:**

We systematically searched PubMed, EMBASE, and Cochrane Central until December 2024 for randomized controlled trials (RCTs) and observational studies assessing changes in left ventricular ejection fraction (LVEF), N-terminal pro-B-type natriuretic peptide (NT-proBNP) levels, systolic blood pressure (SBP), left atrial diameter (LAD), and left ventricular end-diastolic dimension (LVDd) with sacubitril/valsartan use in PD patients. Safety endpoints included hyperkalemia, hypotension, and angioedema. Statistical analyses were performed in R, using proportions for binary and mean differences (MDs) for continuous outcomes.

**Results:**

Nine studies were included, comprising 8 observational studies and 1 RCT, involving 343 PD patients. LVEF improved significantly (MD 5.22; 95% CI, 3.86 to 6.58; p < 0.0001; I^2^ = 38.9%). Sacubitril/valsartan reduced NT-proBNP levels (MD –5630.40; 95% CI, –9177.57 to –2083.23; p = 0.0019; I^2^ = 86%) and SBP (MD –14.59; 95% CI, –20.59 to –8.59; p < 0.0001; I^2^ = 93.5%). No statistically significant changes were noted in LAD (p = 0.0561) or LVDd (p = 0.1037). Hypotension and angioedema were rare events, whereas hyperkalemia showed a slight increase (11.94%).

**Conclusion:**

Sacubitril/valsartan was associated with improvements in cardiac function surrogates and blood pressure in PD patients with HF, with an overall acceptable safety profile despite a modest increase in hyperkalemia. These findings suggest potential benefit in this understudied population, though confirmation in adequately powered RCTs remains necessary.

## Introduction

Chronic kidney disease (CKD) represents a growing global health burden and is currently responsible for over one million deaths annually^1^. Among patients with end-stage kidney disease (ESKD) requiring dialysis, mortality rates remain disproportionately high compared with the general population, with cardiovascular disease (CVD) being the leading cause of death^2,3^. Heart failure (HF) is the most prevalent CVD in this population, and its incidence rises in parallel with the progressive decline in kidney function. In the United States, approximately 25% of patients receiving dialysis are affected by HF^4,5,6^. Patients with advanced CKD and concomitant HF represent a particularly high-risk subgroup with limited therapeutic options, especially those who are ineligible for newer pharmacologic agents due to safety concerns or lack of supporting evidence^7^.

Peritoneal dialysis (PD) serves as a physiologically favorable modality for managing cardiorenal syndrome (CRS), a condition defined by the bidirectional dysfunction of the heart and kidneys^8^. PD offers several theoretical advantages in this setting: it enables gentle and continuous ultrafiltration, preserves residual kidney function more effectively than intermittent hemodialysis (HD), facilitates daily control of electrolytes and volume status, reduces intra-abdominal pressure (a contributor to kidney congestion in right-sided HF), and can be performed at home^9^. These features may, in turn, enhance the tolerability and potential efficacy of HF therapies by creating a more favorable hemodynamic and metabolic milieu^10,11,12,13,14,15^.

Sacubitril/valsartan, the first angiotensin receptor–neprilysin inhibitor (ARNI), has emerged in recent years as a cornerstone in the treatment of heart failure with reduced ejection fraction (HFrEF)^16^. Large-scale randomized controlled trials (RCTs) have demonstrated that sacubitril/valsartan significantly reduces cardiovascular mortality and HF-related hospitalizations compared with conventional renin– angiotensin system inhibitors^17,18^. Consequently, its use has been incorporated as a class I recommendation in major international cardiology guidelines^19,20^. Despite this rationale, the use of sacubitril/valsartan in patients on PD remains understudied^4,7^.

To provide a comprehensive overview of the existing literature, we conducted a systematic review and single-arm meta-analysis to assess the efficacy and safety of sacubitril/valsartan in patients receiving PD. Our analysis focused on changes in echocardiographic parameters, N-terminal pro-B-type natriuretic peptide (NT-proBNP) levels, blood pressure, and the incidence of adverse events.

## Methods

### Eligibility Criteria

Studies eligible for inclusion in this systematic review and meta-analysis were required to meet the following criteria: (1) RCTs or observational studies; (2) evaluation of the efficacy and safety of sacubitril/valsartan; (3) inclusion of patients undergoing PD; and (4) reporting of at least one of the predefined outcomes of interest. We excluded studies based on the following criteria: (1) those combining data from both HD and PD patients; and (2) studies that did not report any relevant outcomes. In cases where multiple studies reported data from overlapping patient populations, only the study with the largest sample size was retained for analysis.

### Search Strategy and data Extraction

We conducted a systematic search of PubMed, EMBASE, and the Cochrane Central Register of Controlled Trials from inception through December 2024, without language restrictions. The search strategy incorporated the following terms: “peritoneal dialysis,” “valsartan,” “sacubitril,” “Entresto,” “ARNI,” “neprilysin inhibitor,” and “LCZ696.” The complete search strategies for each database are detailed in the Supplementary Appendix. In addition, the reference lists of all included articles were manually screened to identify potentially eligible studies not captured through database searches.

Data extraction was performed independently by two reviewers (C.L. and P.E.) using pre-established eligibility criteria and quality assessment protocols. Discrepancies were resolved by consensus with the involvement of a third author (M.G.). This systematic review was prospectively registered in the International Prospective Register of Systematic Reviews (PROSPERO) under registration number CRD42025643186, in accordance with recommended reporting practices.

### Endpoints

The primary efficacy outcomes were the absolute changes from baseline in (1) left ventricular ejection fraction (LVEF) and (2) NT-proBNP levels. Secondary efficacy outcomes included changes in (1) systolic blood pressure (SBP), (2) left atrial diameter (LAD), and (3) left ventricular end-diastolic dimension (LVDd). In addition, predefined safety outcomes were the incidence of (1) hyperkalemia, (2) hypotension, and (3) angioedema during follow-up.

### Quality Assessment

Risk of bias was assessed using appropriate tools according to study design. For RCTs, we applied the Cochrane Collaboration’s Risk of Bias 2.0 (RoB 2) tool; for observational studies with a control group, we used the Risk of Bias in Non-randomized Studies of Interventions (ROBINS-I); and for single-arm studies, the Joanna Briggs Institute (JBI) Critical Appraisal Checklist for Case Series was employed^21,22,23^. The RoB 2 tool evaluates five domains, classifying studies as having “low risk,” “some concerns,” or “high risk” of bias. The ROBINS-I tool assesses seven domains, assigning ratings of “low,” “moderate,” “serious,” or “critical” risk of bias, or indicating “no information.” The JBI tool comprises 10 items with binary (“yes” or “no”) responses, with higher cumulative scores reflecting a lower overall risk of bias. Two authors (C.L. and P.E.) independently performed the assessments, and any disagreements were resolved through discussion with a third author.

Given the small number of included studies, a formal evaluation of publication bias was not feasible. Funnel plots are not reliable for detecting asymmetry in meta-analyses with fewer than 10 studies, and the Egger test is similarly underpowered in such settings^24^.

### Statistical Analysis

This systematic review and meta-analysis was conducted and reported in accordance with the methodological guidance outlined in the Cochrane Handbook for Systematic Reviews of Interventions and adhered to the Preferred Reporting Items for Systematic Reviews and Meta-Analyses (PRISMA) statement^25,26^.

For continuous outcomes, we calculated the mean difference (MD) from baseline with corresponding 95% confidence intervals (CIs). Statistical significance was defined as a two-tailed p-value < 0.05. Pooled analyses for continuous variables were conducted using a random-effects model with the restricted maximum likelihood (REML) estimator. Safety outcomes were summarized descriptively in a table reporting the number of adverse events, the total number of patients exposed, the number of studies reporting each event, and the corresponding proportion.

Heterogeneity among studies was evaluated using the Cochran Q test and quantified with the I^2^ statistic; an I^2^ value > 50% was considered indicative of substantial heterogeneity. Sensitivity analyses were performed using a leave-one-out approach to determine the influence of individual studies on the overall pooled estimates. All statistical analyses were conducted using R software (version 4.4.2; R Foundation for Statistical Computing, Vienna, Austria).

## Results

**Figure 1:**
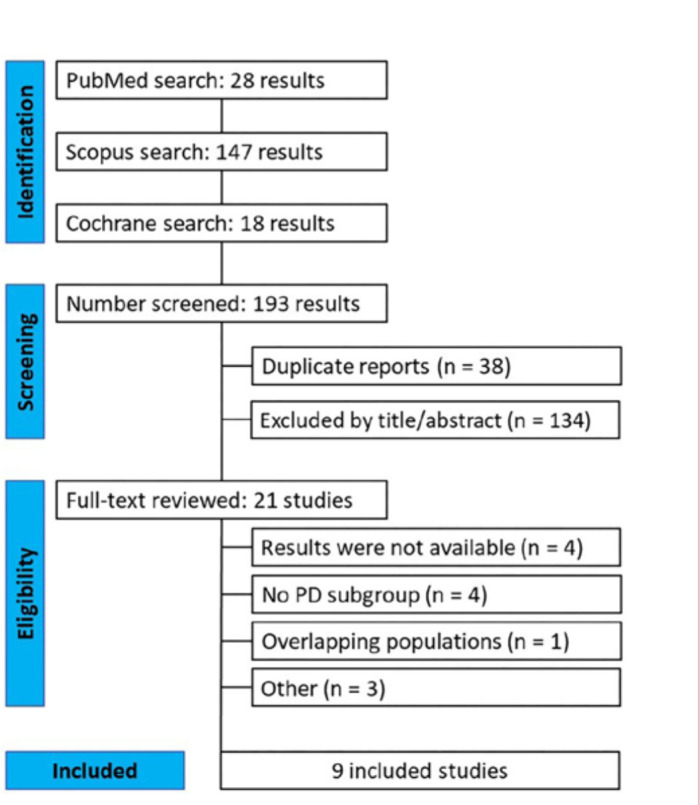
PRISMA flow diagram of study screening and selection.

As summarized in Figure 1, a total of 193 records were identified through systematic searches of three databases. After removal of duplicates and screening for relevance, 21 full-text articles were assessed for eligibility. Of these, nine studies met the inclusion criteria and were included in the final analysis, comprising a total of 343 patients with HF undergoing PD while receiving sacubitril/valsartan therapy^27,28,29,30,31,32,33,34,35^.

Baseline characteristics of the studies are detailed in Table 1. The included studies employed either continuous ambulatory peritoneal dialysis (CAPD) or automated peritoneal dialysis (APD) as the dialysis modality. Sacubitril/valsartan was prescribed at varying doses across studies, reflecting real-world clinical practice. Follow-up durations ranged from three to twelve months, and the mean age of participants varied from 45.3 to 71 years.

**Table 1:** Baseline characteristics of the studies

Study	Study design	Total patients on PD	SV group	Male, n (%)	Mean age (years)	PD modality	SV dose	Follow-up
Ding 2023	Retrospective cohort study	64	32	NA	NA	CAPD or APD	50–100 mg, BID	Median 349 days
Fu 2021	Retrospective single-arm study	21	21	14 (66.6)	51	CAPD	50–100 mg, BID	3 to 12 months
He 2023	Cross-sectional study	40	40	18 (45)	45.3	CAPD or APD	3 groups: 50 mg, BID, 100 mg, QD or 100 mg – based on BP	4 months
Ma 2023	Retrospective cohort study	99	61	44 (72.1)	52	CAPD or APD	50–100 mg, BID	6 to 12 months
Niu 2022	Case-control study	16	10	NA	NA	NA	From 24/26 mg, BID to 97/103 mg, BID	12 months
Pimenta 2023	Cross-sectional cohort study	5	5	5 (100)	71	CAPD	24/26 mg, BID	Median 16 months
Sheng 2023	RCT	160	80	64 (40)	57.6	CAPD	50–100 mg, BID	6 months
Wang 2024	Retrospective cohort study	102	47	63 (61.7)	56.8	CAPD	NA	12 months
Zhang 2022	Retrospective single-arm study	47	47	28 (59.5)	45.9	CAPD or APD	100 mg, BID	7 days

Abbreviations – PD: peritoneal dialysis, SV: sacubitril-valsartan, NA: not available, CAPD: continuous ambulatory peritoneal dialysis, APD: automated peritoneal dialysis, BID: twice daily, QD: once daily.

**Figure 2:**
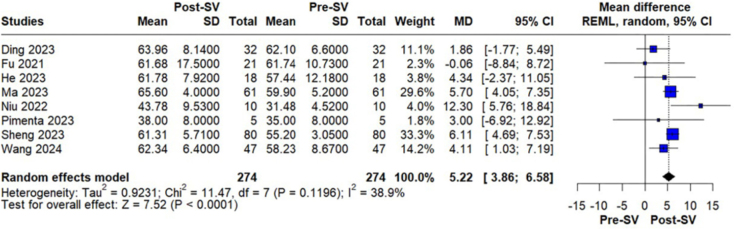
Changes in LVEF in PD patients before and after sacubitril/valsartan treatment.

**Figure 3:**
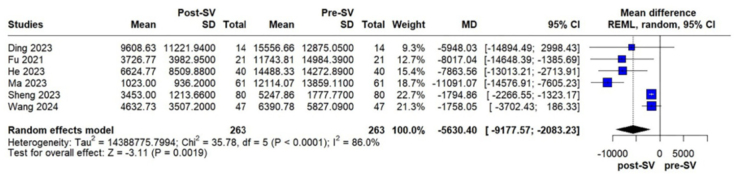
Changes in NT-proBNP levels in PD patients before and after sacubitril/valsartan treatment.

**Figure 4:**
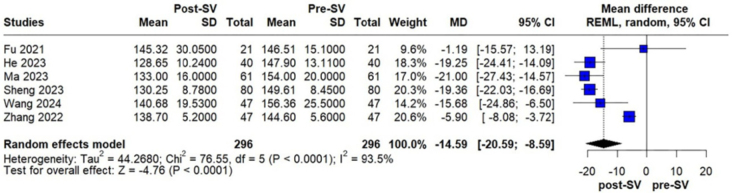
Changes in SBP in PD patients before and after sacubitril/valsartan treatment.

Treatment with sacubitril/valsartan was associated with a statistically significant improvement in LVEF, with a pooled MD of 5.22% (95% CI, 3.86 to 6.58; p < 0.0001; I^2^ = 38.9%; Figure 2). A significant reduction in NT-proBNP levels was also observed (MD –5630.40 pg/mL; 95% CI, –9177.57 to –2083.23; p = 0.0019; I^2^ = 86%; Figure 3). Furthermore, SBP was significantly decreased following treatment (MD –14.59 mmHg; 95% CI, –20.59 to –8.59; p < 0.0001; I^2^ = 93.5%; Figure 4). In contrast, changes in LAD and LVDd did not reach statistical significance. The pooled MD for LAD was –3.31 mm (95% CI, –6.70 to 0.09; p = 0.0561; I^2^ = 91.4%; Figure S1), and the pooled MD for LVDd was –4.46 mm (95% CI, –9.83 to 0.91; p = 0.1037; I^2^ = 94.7%; Figure S2), indicating substantial heterogeneity and inconclusive effects on structural remodeling.

Abbreviations – CI, confidence interval; LVEF, left ventricular ejection fraction; MD, mean difference; PD, peritoneal dialysis; REML, restricted maximum likelihood; SD, standard deviation; SV, sacubitril/valsartan.

Abbreviations – CI, confidence interval; MD, mean difference; NT-proBNP, N-terminal pro-B-type natriuretic peptide; PD, peritoneal dialysis; REML, restricted maximum likelihood; SD, standard deviation; SV, sacubitril/valsartan.

Abbreviations – CI, confidence interval; MD, mean difference; PD, peritoneal dialysis; REML, restricted maximum likelihood; SBP, systolic blood pressure; SD, standard deviation; SV, sacubitril/valsartan.

Regarding safety outcomes, a modest incidence of hyperkalemia was observed following sacubitril/valsartan initiation, with 16 events among 134 patients (11.9%; Table 2). Hypotension occurred infrequently, with only 3 events reported among 206 patients (1.5%). Notably, no cases of angioedema were identified across six studies reporting on this outcome, encompassing a total of 211 patients receiving PD.

**Table 2:** Adverse events related to the use of sacubitril/valsartan

Outcome	Number of studies reporting	Total of events	Total number of patients	Proportion (%)
Hypotension	6	3	206	3/206 (1.45%)
Hyperkalemia	4	16	134	16/134 (11.94%)
Angioedema	6	0	211	0/211 (0%)

Leave-one-out sensitivity analyses were performed by sequentially excluding each study from the pooled estimates. The primary efficacy outcomes — MD from baseline in LVEF, NT-proBNP levels, and SBP — remained statistically significant across all iterations, supporting the robustness of these findings. In contrast, the pooled results for LVDd and LAD exhibited greater variability upon removal of individual studies, indicating limited stability and potential sensitivity to study-level characteristics. Each of these individual analyses can be found in the Supplementary Material. The sensitivity analysis also highlighted that certain studies substantially contributed to the high heterogeneity observed across several outcomes.

The individual evaluation of each study included in the meta-analysis is presented in the Supplementary Material. The only RCT was found to have a risk of bias classified as “some concerns.” The observational studies were classified as having a “serious” or “moderate” risk of bias with the ROBINS-I tool and as “low” or “moderate” risk with the JBI appraisal tool.

## Discussion

In this systematic review and meta-analysis of nine studies, we evaluated the efficacy and safety of sacubitril/valsartan in a cohort of 343 patients with HF undergoing PD. The main findings were as follows: (1) sacubitril/valsartan was associated with a significant improvement in LVEF (MD 5.22%; p < 0.0001) and a substantial reduction in serum NT-proBNP levels (MD –5630.40 pg/mL; p = 0.0019); (2) a significant decrease in SBP was observed following treatment (MD –14.59 mmHg; p < 0.0001); and (3) no statistically significant changes were noted in LAD or LVDd. Regarding safety outcomes, hypotension and angioedema events were rare, while a modest incidence of hyperkalemia (11.94%) was reported among patients receiving sacubitril/valsartan.

The PARADIGM-HF trial represented a landmark in the development of ARNI therapy. This pivotal study demonstrated that sacubitril/valsartan significantly reduced the risk of cardiovascular mortality and hospitalization for HF while also improving symptoms and physical limitations in patients with HFrEF when compared with enalapril^17^. Subsequent trials further reinforced the superiority of sacubitril/valsartan over both angiotensin-converting enzyme inhibitors (ACEis) and angiotensin II receptor blockers (ARBs), particularly in lowering NT-proBNP levels and reducing left ventricular mass^18,36,37^. However, a critical limitation of these trials is the systematic exclusion of patients with ESKD, resulting in a persistent gap in the evidence base regarding the safety and efficacy of ARNI therapy in this high-risk population.

Recently, two meta-analyses involving patients with ESKD on dialysis were conducted to address this evidence gap. These studies demonstrated that sacubitril/valsartan was associated with improvements in left ventricular function and structure, as well as a reduction in all-cause mortality, without a significant increase in adverse events^38,39^. However, both analyses combined data from patients on HD and PD, with the majority of participants undergoing HD. This is a critical limitation, as the management of CVD in PD patients differs substantially from that in patients on HD due to variations in fluid removal strategies, dialysate composition, and residual kidney function. In PD, glucose-based solutions, continuous ultrafiltration, potassium homeostasis, and preservation of volume status are key components of cardiovascular management^3^. To overcome these limitations, our meta-analysis focused exclusively on patients undergoing PD, allowing for a more targeted evaluation of sacubitril/valsartan in this specific population and yielding more precise and clinically relevant conclusions regarding its efficacy and safety.

In patients undergoing dialysis, deleterious remodeling of LV structure and function is strongly associated with increased risks of cardiovascular morbidity and mortality^5^. One of the primary therapeutic goals of ARNI therapy is to counteract this adverse remodeling by reducing cardiac volumes and improving LV function^40^. A recent meta-analysis involving over 10,000 patients demonstrated that ARNIs improved echocardiographic indices—including LVEF, LV volumes, and measures of hypertrophy^41^. In line with these findings, our metaanalysis observed a significant improvement in LVEF from baseline in PD patients treated with sacubitril/valsartan. However, no statistically significant changes were detected in other structural echocardiographic parameters, such as LAD and LVDd. A potential explanation for this divergence lies in the heterogeneity of the study populations. Among the studies reporting LAD and LVDd, three out of four included patients with a broad range of LVEF phenotypes. This clinical heterogeneity, reflecting varying pathophysiological mechanisms and therapeutic responsiveness, may contribute to the attenuated or inconsistent effects of sacubitril/valsartan on structural cardiac parameters beyond LVEF^42^. Additionally, structural remodeling may be observed over a longer follow-up period than in the individual studies included in these analyses due to confounding factors, such as decreased volume overload and improved blood pressure control with PD^43^.

Among patients on PD, elevated levels of NT-proBNP have been associated with adverse cardiac remodeling, including left ventricular hypertrophy and coronary artery disease^40,44^. Sacubitril/valsartan, through its reverse remodeling properties, has been shown to lower circulating natriuretic peptide concentrations, contributing to improved clinical outcomes^40^. In our meta-analysis, we observed a significant reduction in NT-proBNP following treatment, reinforcing the drug’s mechanistic role in improving cardiac structure and function. These findings suggest its potential utility in tracking disease progression in PD patients. However, due to the influence of reduced kidney clearance on peptide accumulation, its reliability in the diagnostic and prognostic evaluation of HF in dialysis populations remains limited^5,39,44^. Accordingly, current guidelines do not recommend the routine use of NT-proBNP for HF evaluation in patients receiving dialysis.

Some adverse events associated with ARNI use in patients with kidney dysfunction may be linked to altered pharmacokinetics. After administration, sacubitril is converted to its active metabolite, sacubitrilat, which is primarily cleared by the kidneys, whereas valsartan is eliminated via the biliary route. As such, kidney impairment is expected to influence the pharmacokinetics of sacubitrilat but not that of valsartan^45,46^. A recent study evaluated sacubitrilat levels in PD patients and found reduced urinary excretion compared with healthy individuals. PD appeared to have only a limited capacity to remove sacubitrilat from the circulation, likely due to the drug’s high plasma protein binding. Despite these pharmacokinetic changes, overall exposure to sacubitrilat in PD patients remained within the therapeutic range observed in the general population, suggesting that a 100 mg twice-daily dosing regimen is likely safe and does not require adjustment. This safety profile was further supported by the absence of significant adverse events in the 40 patients included in the study^29^.

The main strength of this systematic review lies in its exclusive focus on PD patients, a population normally underrepresented in clinical trials evaluating the use of sacubitril/valsartan. In addition, the consistency of results across sensitivity analyses reinforces the robustness of our findings. Nonetheless, our study has several limitations. First, eight of the nine included studies were observational in nature, which may introduce inherent biases and limit causal inference. Second, there was a substantial variability in LVEF, baseline population characteristics, sacubitril/valsartan dosing regimens, and duration of follow-up across studies. These differences contributed to the moderate-to-high heterogeneity observed in many of the pooled outcomes. Although sensitivity analyses did not reveal significant influence from individual studies, the results should nonetheless be interpreted with caution. Third, critical clinical outcomes—such as cardiovascular mortality, hospitalization rates, and all-cause mortality—could not be evaluated due to limited reporting. Lastly, the majority of studies (8 out of 9) were conducted in Asian countries, which may restrict the generalizability of our findings to broader and more diverse patient populations. Despite these limitations, our study is the first meta-analysis evaluating the benefit of sacubitril/valsartan in PD patients. Considering that patients with kidney failure have a higher risk of developing HF, an optimized treatment for those correctly diagnosed is imperative to improve patient survival and quality of life.

## Conclusion

In conclusion, this systematic review and metaanalysis suggests that sacubitril/valsartan may offer clinical benefits for patients on PD. The pooled evidence indicates improvements in LVEF, reductions in NT-proBNP levels, and enhanced blood pressure control, without a corresponding increase in adverse events. However, the predominance of observational data and the significant variation across studies pose important limitations and demand cautious interpretation. Despite these constraints, sacubitril/valsartan remains a promising therapeutic option in this high-risk population. Further high-quality RCTs are warranted to validate its efficacy and safety in patients undergoing PD.

## Data Availability

The datasets generated and/or analyzed during the current study are available from the corresponding author upon reasonable request and are also available in the manuscript tables and figures.

## Supplementary Material

The following online material is available for this article:

Supplementary material – Search strategy.
