# Supplementary material for: Efficacy and safety of sacubitril/valsartan in patients on peritoneal dialysis: a systematic review and meta-analysis
Source: J Bras Nefrol. 2026 Mar 30;48(3):e20250318. doi: 10.1590/2175-8239-JBN-2025-0318en (PMC13051583; doi:10.1590/2175-8239-JBN-2025-0318en)
Supplement: Supplementary file 1 [file 2175-8239-jbn-48-3-e20250318-suppl1.pdf]

Braz. J. Nephrol.
https://doi.org/10.1590/2175-8239-JBN-2025-0318en

Supplementary Material to "Efficacy and safety of sacubitril/valsartan in patients on peritoneal dialysis: a systematic review and meta-analysis"

Search strategy

Pubmed: ("Peritoneal Dialysis"[Mesh] OR "Peritoneal dialysis") AND (Valsartan OR sacubitril OR entresto OR ARNI OR "neprilysin inhibitor" OR LCZ696)

Embase: ('peritoneal dialysis'/exp OR 'peritoneal dialysis') AND ('valsartan' OR 'sacubitril' OR 'entresto' OR 'arni' OR 'neprilysin inhibitor' OR 'lcz696')

Cochrane: ("Peritoneal dialysis") AND (Valsartan OR sacubitril OR entresto OR ARNI OR "neprilysin inhibitor" OR LCZ696)

Pooled analysis of echocardiographic parameters

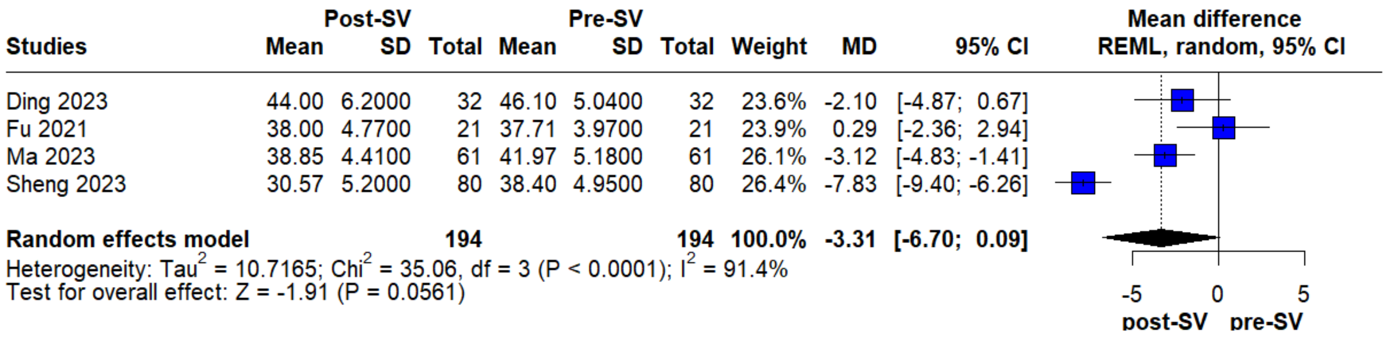

Figure S1 - Changes in LAD in PD patients before and after sacubitril/valsartan treatment. CI, confidence interval; LAD, left atrial diameter; MD, mean difference; PD, peritoneal dialysis; REML, restricted maximum likelihood; SD, standard deviation; SV, sacubitril/valsartan.

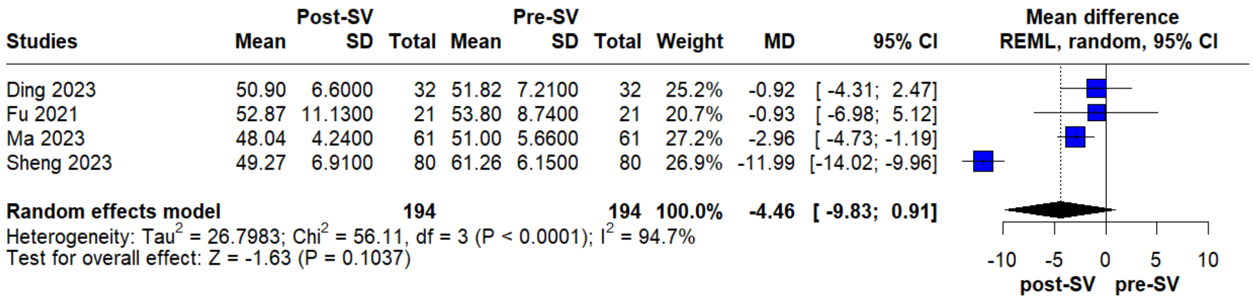

Figure S2 - Changes in LVDd in PD patients before and after sacubitril/valsartan treatment. CI, confidence interval; LVDd, left ventricular end-diastolic dimension; MD, mean difference; PD, peritoneal dialysis; REML, restricted maximum likelihood; SD, standard deviation; SV, sacubitril/valsartan.

Leave-one-out Forrest Plots for each individual outcome

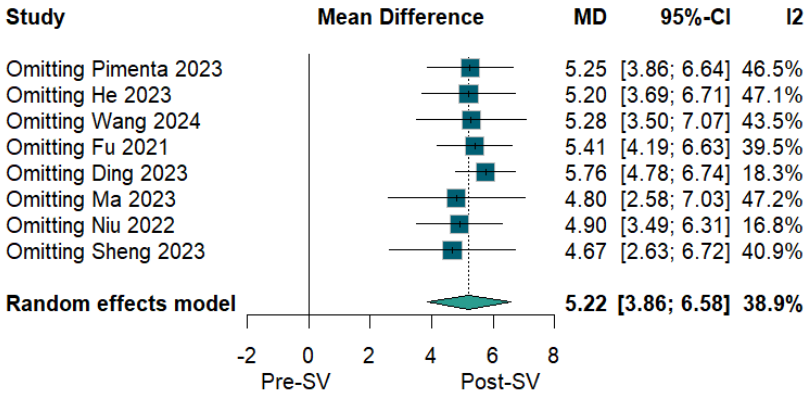

Figure S3 - Leave-one-out analyses for the outcome of LVEF.

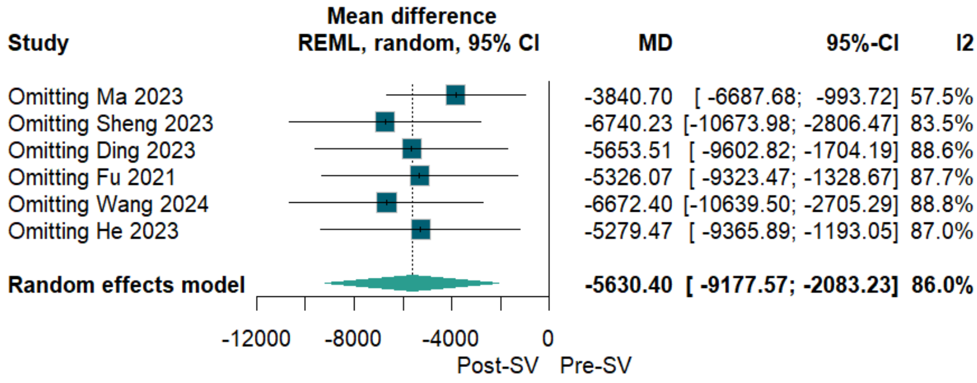

Figure S4 - Leave-one-out analyses for the outcome of NT-proBNP.

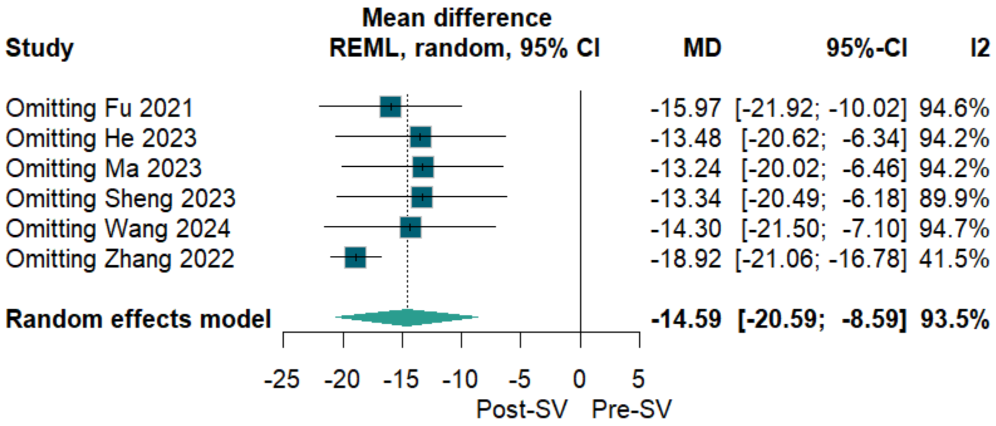

Figure S5. Leave-one-out analyses for the outcome of SBP.

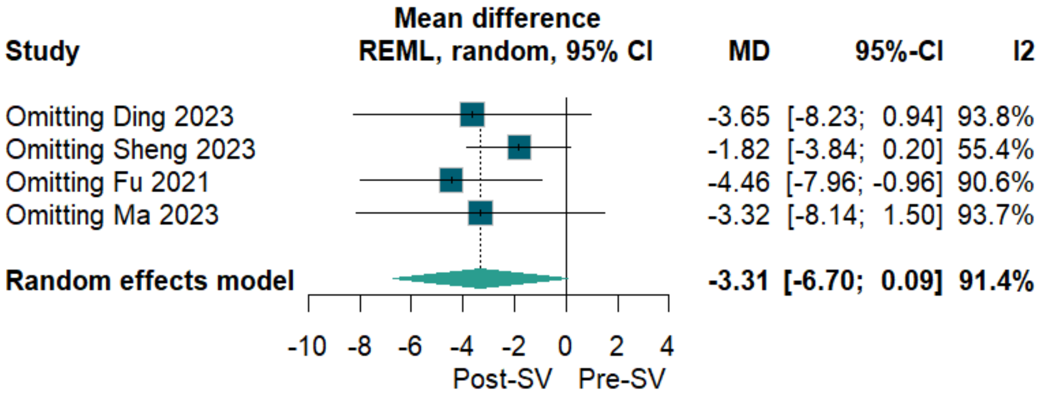

Figure S6. Leave-one-out analyses for the outcome of LAD.

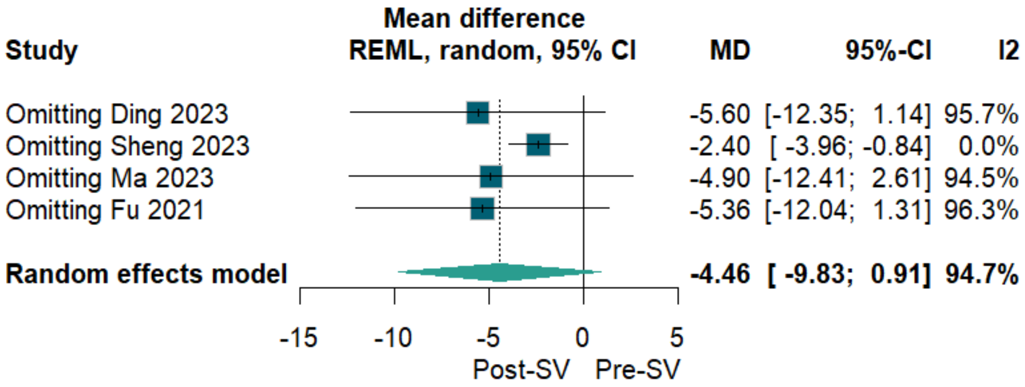

Figure S7. Leave-one-out analyses for the outcome of LVDd.

Risk of bias summary for randomized studies (RoB 2)

Study	Bias from randomization process	Bias due to deviations from intended interventions	Bias due to missing outcome data	Bias in measurement of the outcomes	Bias in selection of the reported result	Overall risk of bias
Sheng 2023	Some concerns	Low	Low	Low	Some concerns	Some concerns

Risk of bias summary for non-randomized studies (ROBINS-I)

Study	Bias due to confounding	Bias in selection of participants	Bias in classification of interventions	Bias due to deviations from intended interventions	Bias due to missing data	Bias in measurement of outcomes	Bias in selection of the reported result	Overall risk of bias judgement
Ding 2023	Low	Moderate	Moderate	Low	Low	Moderate	Low	Moderate
Ma 2023	Serious	Moderate	Moderate	Moderate	Low	Low	Moderate	Serious
Niu 2022	Serious	Low	Low	Low	Low	Low	Moderate	Serious
Wang 2024	Low	Moderate	Serious	Low	Moderate	Moderate	Low	Serious

JBI Critical Appraisal Checklists for Case series Studies

**JBI Critical Appraisal Checklists for Case series Studies**	Fu 2021	He 2023	Pimenta 2023	Zhang 2022
Were there clear criteria for inclusion in the case series?	Yes	Yes	Yes	Yes
Was the condition measured in a standard, reliable way for all participants included in the case series?	Yes	Yes	No	Yes
Were valid methods used for identification of the condition for all participants included in the case series?	No	Unclear	No	Unclear
Did the case series have consecutive inclusion of participants?	Yes	Yes	No	Yes
Did the case series have complete inclusion of participants?	Unclear	No	Unclear	Yes
Was there clear reporting of the demographics of the participants in the study?	Yes	Yes	Yes	Yes
Was there clear reporting of clinical information of the participants?	Yes	Yes	Yes	Yes
Were the outcomes or follow up results of cases clearly reported?	Yes	Yes	Yes	Yes

Was there clear reporting of the presenting site(s)/clinic(s) demographic information?	No	No	No	No
Was statistical analysis appropriate?	Yes	Yes	No	Yes
**Total quality assessment score for each study**	70%	70%	40%	80%
