# Supplementary material for: Efficacy and safety of sacubitril/valsartan in patients on peritoneal dialysis: a systematic review and meta-analysis
Source: J Bras Nefrol. 2026 Mar 30;48(3):e20250318. doi: 10.1590/2175-8239-JBN-2025-0318en (PMC13051583; doi:10.1590/2175-8239-JBN-2025-0318en)
Supplement: Supplementary file 2 [file 2175-8239-jbn-48-3-e20250318-suppl2.pdf]

Braz. J. Nephrol.
https://doi.org/10.1590/2175-8239-JBN-2025-0318pt

Material Suplementar para "Eficácia e segurança do sacubitril/valsartana em pacientes em diálise peritoneal: uma revisão sistemática e meta-análise"

Estratégia de busca

Pubmed: ("Peritoneal Dialysis"[Mesh] OR "Peritoneal dialysis") AND (Valsartan OR sacubitril OR entresto OR ARNI OR "neprilysin inhibitor" OR LCZ696)

Embase: ('peritoneal dialysis'/exp OR 'peritoneal dialysis') AND ('valsartan' OR 'sacubitril' OR 'entresto' OR 'arni' OR 'neprilysin inhibitor' OR 'lcz696')

Cochrane: ("Peritoneal dialysis") AND (Valsartan OR sacubitril OR entresto OR ARNI OR "neprilysin inhibitor" OR LCZ696)

Análise estatística dos parâmetros ecocardiográficos

	Pós-SV			Pré-SV						Mean difference
**Estudos**	**Média**	**SD**	**Total**	**Média**	**SD**	**Total**	**Weight**	**MD**	**95% CI**	**REML, random, 95% CI**
Ding 2023	44.00	6.2000	32	46.10	5.0400	32	23.6%	-2.10	[-4.87; 0.67]	
Fu 2021	38.00	4.7700	21	37.71	3.9700	21	23.9%	0.29	[-2.36; 2.94]	
Ma 2023	38.85	4.4100	61	41.97	5.1800	61	26.1%	-3.12	[-4.83; -1.41]	
Sheng 2023	30.57	5.2000	80	38.40	4.9500	80	26.4%	-7.83	[-9.40; -6.26]	
**Modelo de efeitos randômicos**			**194**			**194**	**100.0%**	**-3.31**	**[-6.70; 0.09]**	

Heterogeneity: $Tau^2 = 10.7165$; $Chi^2 = 35.06$, df = 3 (P < 0.0001); $I^2 = 91.4\%$
Test for overall effect: Z = -1.91 (P = 0.0561)

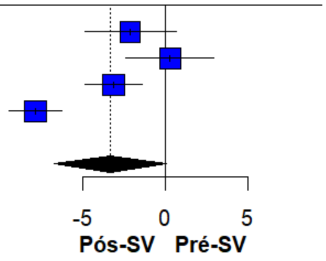

Figura S1 - Alterações no DAE em pacientes em DP antes e após o tratamento com sacubitril/valsartana. CI, intervalo de confiança; DAE, diâmetro do átrio esquerdo; DM, mean difference; DP, diálise peritoneal; REML, restricted maximum likelihood; SD, desvio padrão; SV, sacubitril/valsartana.

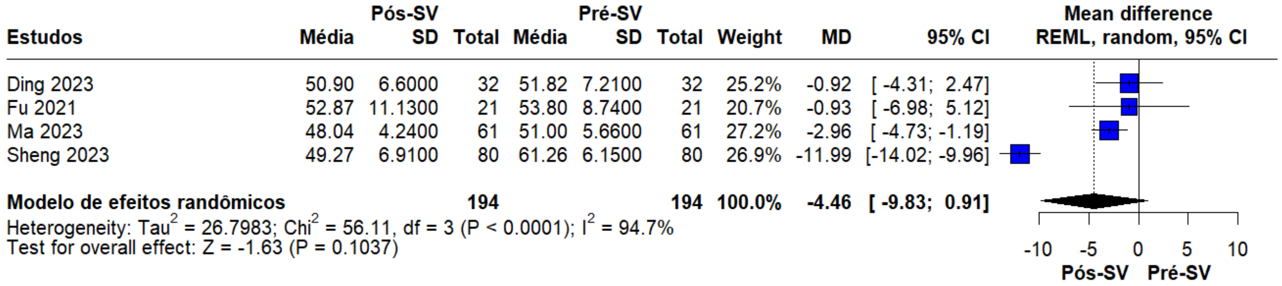

Figura S2 - Alterações na DDVE em pacientes em DP antes e após o tratamento com sacubitril/valsartana. CI, intervalo de confiança; DDVE, diâmetro diastólico final do ventrículo esquerdo; DM, mean difference; DP, diálise peritoneal; REML, restricted maximum likelihood; SD, desvio padrão; SV, sacubitril/valsartana.

Gráficos da floresta (florest plot) para análises "Leave-one-out" de cada desfecho

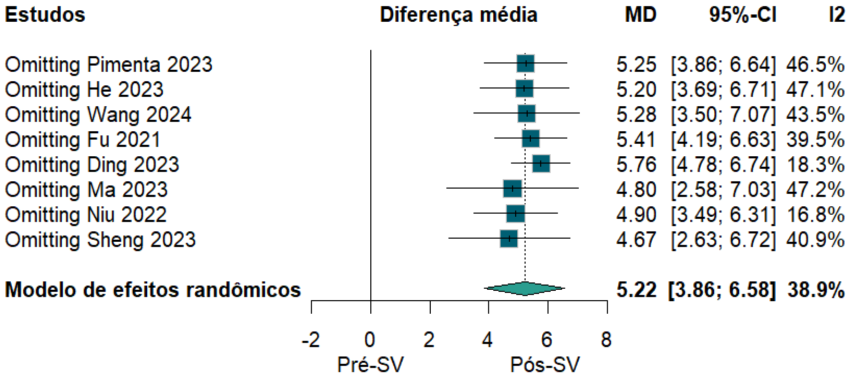

Figura S3 - Análises *leave-one-out* para o desfecho de FEVE.

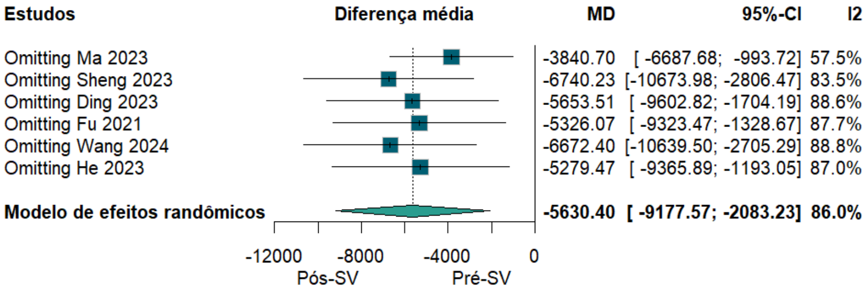

Figura S4 - Análises *leave-one-out* para o desfecho de NT-proBNP.

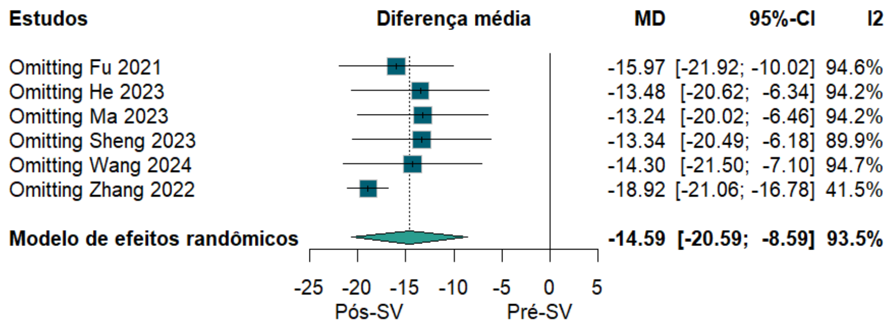

Figura S5 - Análises *leave-one-out* para o desfecho de PAS.

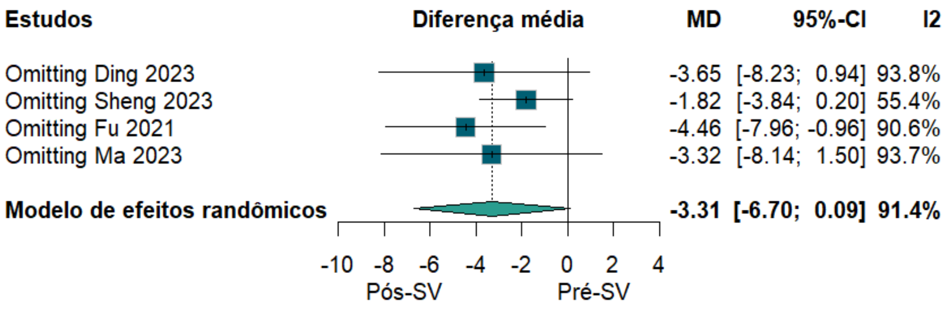

Figura S6 - Análises *leave-one-out* para o desfecho de DAE.

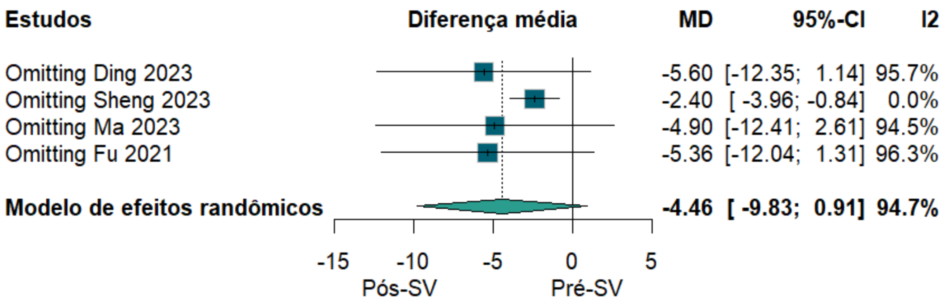

Figura S7 - Análises *leave-one-out* para o desfecho de DDVE.

Resumo do risco de viés para estudos randomizados (RoB 2)

Estudos	Viés no processo de randomização	Viés devido a desvios das intervenções pretendidas	Viés devido a dados faltantes de desfechos	Viés na medição dos desfechos	Viés na seleção do resultado relatado	Risco de viés geral
Sheng 2023	Algumas preocupações	Baixo	Baixo	Baixo	Algumas preocupações	Algumas preocupações

Resumo do risco de viés para estudos não randomizados (ROBINS-I)

Study	Viés por fatores de confudimento	Viés na seleção dos participantes	Viés na classificação das intervenções	Viés devido a desvios das intervenções pretendidas	Viés devido a dados faltantes	Viés na medição dos desfechos	Viés na seleção do resultado relatado	Risco de víes geral
Ding 2023	Baixo	Moderado	Moderado	Baixo	Baixo	Moderado	Baixo	Moderado
Ma 2023	Elevado	Moderado	Moderado	Moderado	Baixo	Baixo	Moderado	Elevado
Niu 2022	Elevado	Baixo	Baixo	Baixo	Baixo	Baixo	Moderado	Elevado
Wang 2024	Baixo	Moderado	Elevado	Baixo	Moderado	Moderado	Baixo	Elevado

Checklists de Avaliação Crítica do JBI para Séries de Casos

**Checklists de Avaliação Crítica do JBI para Séries de Casos**	Fu 2021	He 2023	Pimenta 2023	Zhang 2022
Havia critérios claros para inclusão na série de casos?	Sim	Sim	Sim	Sim
A condição foi medida de forma padronizada e confiável para todos os participantes incluídos?	Sim	Sim	Não	Sim
Foram utilizados métodos válidos para identificação da condição em todos os participantes?	Não	Incerto	Não	Incerto
A série de casos teve inclusão consecutiva de participantes?	Sim	Sim	Não	Sim
A série de casos teve inclusão completa de participantes?	Incerto	Não	Incerto	Sim
Houve um relato claro da demografia dos participantes no estudo?	Sim	Sim	Sim	Sim
Houve um relato claro das informações clínicas dos participantes?	Sim	Sim	Sim	Sim
Os desfechos ou resultados de acompanhamento (*follow-up*) foram claramente relatados?	Sim	Sim	Sim	Sim
Houve um relato claro das informações demográficas dos locais de atendimento/clínicas?	Não	Não	Não	Não
A análise estatística foi apropriada?	Sim	Sim	Não	Sim

**Pontuação total de avaliação de qualidade para cada estudo**	70%	70%	40%	80%
