# Supplementary material for: Efficacy and safety of sacubitril/valsartan in patients on peritoneal dialysis: a systematic review and meta-analysis
Source: J Bras Nefrol. 2026 Mar 30;48(3):e20250318. doi: 10.1590/2175-8239-JBN-2025-0318en (PMC13051583; doi:10.1590/2175-8239-JBN-2025-0318en)
Supplement: Translation

# Translation: Eficácia e segurança do sacubitril/valsartana em pacientes em diálise peritoneal: uma revisão sistemática e meta-análise

**DOI:** 10.1590/2175-8239-JBN-2025-0318pt

**Authors:** Caio Lima da Silva, Pandora Eloa Oliveira Fonseca, Viviane Calice-Silva, Daniela Ponce, Paulo Novis Rocha, Maria Gabriela Guimarães

**Affiliations:** 1 Universidade Federal do Rio Grande do Norte, Natal, RN, Brasil.; 2 Universidade Federal de Campina Grande, Campina Grande, PA, Brasil.; 3 Fundação Pró-Rim, Joinville, SC, Brasil.; 4 Universidade de Região de Joinville, Joinville, SC, Brasil.; 5 Universidade do Estado de São Paulo, Departamento de Medicina Clínica, São Paulo, SP, Brasil.; 6 Universidade Federal da Bahia, Departamento de Medicina Clínica, Salvador, BA, Brasil.; 7 Universidade Federal da Bahia, Programa de Pós-Graduação em Ciências da Saúde, Salvador, BA, Brasil.; 8 International Renal Research Institute of Vicenza, Vicenza, Italia.

**Keywords:** Diálise Peritoneal, Sacubitril-Valsartana, Falência Renal Crônica, Insuficiência Cardíaca, Síndrome Cardiorrenal

## Abstract

**Introdução:**

Sacubitril/valsartana é um medicamento recomendado para o manejo da insuficiência cardíaca (IC), mas seu papel em pacientes em diálise peritoneal (DP) ainda permanece incerto. Avaliamos a eficácia e a segurança do sacubitril/valsartana nessa população, por meio desta revisão sistemática e meta-análise de braço único.

**Métodos:**

Realizamos uma busca sistemática no PubMed, EMBASE e Cochrane Central, até dezembro de 2024, por ensaios clínicos randomizados (ECRs) e estudos observacionais que avaliassem alterações na fração de ejeção do ventrículo esquerdo (FEVE), nos níveis do fragmento N-terminal do peptídeo natriurético tipo B (NT-proBNP), na pressão arterial sistólica (PAS), no diâmetro do átrio esquerdo (DAE) e na dimensão diastólica final do ventrículo esquerdo (DDFVE) basais, com o uso de sacubitril/valsartana em pacientes submetidos à DP.

**Resultados:**

Incluímos 9 estudos (8 observacionais, 1 ECR), totalizando 343 pacientes. A FEVE melhorou significativamente (MD 5,22; 95% CI, 3,86 a 6,58; p < 0,0001; I^2^ = 38,9%). O tratamento foi associado a reduções no NT-proBNP (MD –5630,40; IC 95% CI, –9177,57 a –2083,23; p = 0,0019; I^2^ = 86%) e na PAS (MD –14,59; 95% CI, –20,59 a –8,59; p < 0,0001; I^2^ = 93,5%). Não houve mudanças significativas no DAE (p = 0,0561) ou na DDFVE (p = 0,1037). Hipotensão e angioedema foram raros, enquanto a hipercalemia ocorreu em 11,94% dos casos.

**Conclusão:**

O sacubitril/valsartana associou-se a melhorias na função cardíaca e na pressão arterial em pacientes em DP com IC, apresentando perfil de segurança aceitável, apesar do aumento modesto da hipercalemia. Os achados sugerem benefícios nesta população frequentemente excluída de ensaios clínicos, embora a confirmação em ECRs com poder adequado seja necessária.

## Introdução

A doença renal crônica (DRC) representa um crescente problema de saúde global e, atualmente, é responsável por mais de um milhão de mortes anualmente^1^. Entre os pacientes com doença renal terminal (DRT) que necessitam de diálise, as taxas de mortalidade permanecem desproporcionalmente elevadas em comparação com a população geral, sendo as doenças cardiovasculares (DCV) a principal causa de óbito^2,3^. A insuficiência cardíaca (IC) é a DCV mais prevalente nesta população, e sua incidência aumenta paralelamente ao declínio progressivo da função renal. Nos Estados Unidos, aproximadamente 25% dos pacientes em diálise são afetados pela IC^4,5,6^. Pacientes com DRC avançada e IC concomitante representam um subgrupo de alto risco, com opções terapêuticas limitadas, especialmente aqueles inelegíveis para novos agentes farmacológicos devido a preocupações de segurança ou à falta de evidências^7^.

A diálise peritoneal (DP) serve como uma modalidade fisiologicamente favorável para o manejo da síndrome cardiorrenal (SCR), uma condição definida pela disfunção bidirecional do coração e dos rins^8^. A DP oferece diversas vantagens teóricas neste cenário: permite uma ultrafiltração suave e contínua, preserva a função renal residual de forma mais eficaz do que a hemodiálise (HD) intermitente, facilita o controle diário de eletrólitos e do estado volêmico, reduz a pressão intra-abdominal (um fator contribuinte para a congestão renal na IC direita) e pode ser realizada em domicílio^9^. Essas características podem, por sua vez, aumentar a tolerabilidade e a eficácia potencial das terapias para IC ao criar um meio hemodinâmico e metabólico mais favorável^10,11,12,13,14,15^.

O sacubitril/valsartana, o primeiro inibidor da neprilisina e do receptor de angiotensina (INRA), emergiu nos últimos anos como um pilar fundamental no tratamento da insuficiência cardíaca com fração de ejeção reduzida (ICFER)^16^. Ensaios clínicos randomizados (ECRs) de larga escala demonstraram que o sacubitril/valsartana reduz significativamente a mortalidade cardiovascular e as hospitalizações relacionadas à IC em comparação com os inibidores convencionais do sistema renina-angiotensina^17,18^. Seu uso foi incorporado como uma recomendação de classe I nas principais diretrizes internacionais de cardiologia^19,20^. Apesar disso, o uso de sacubitril/valsartana em pacientes em DP ainda permanece pouco estudado^4,7^.

Para fornecer uma visão abrangente da literatura existente, conduzimos uma revisão sistemática e uma meta-análise de braço único, avaliando a eficácia e a segurança do sacubitril/valsartana em pacientes submetidos à DP. Nossa análise concentrou-se em alterações nos parâmetros ecocardiográficos, nos níveis de peptídeo natriurético tipo B N-terminal (NT-proBNP), na pressão arterial e na incidência de eventos adversos.

## Métodos

### Critérios de Elegibilidade

Estudos elegíveis para inclusão nesta revisão sistemática e meta-análise preencheram os seguintes critérios de inclusão: (1) ECRs ou estudos observacionais; (2) avaliação da eficácia e segurança do sacubitril/valsartana; (3) inclusão de pacientes em DP; e (4) análise de um dos desfechos de interesse predefinidos. Estudos foram excluídos com base nos seguintes critérios: (1) combinação de dados de pacientes em HD e DP; e (2) estudos que não reportaram nenhum desfecho relevante. Em casos de mais de um estudo relatar dados de populações de pacientes sobrepostas, apenas o estudo com o maior tamanho amostral foi retido para análise.

### Estratégia de Busca e Extração de Dados

Realizamos uma busca sistemática nas bases de dados PubMed, EMBASE e no *Cochrane Central Register of Controlled Trials*, desde sua criação até dezembro de 2024, sem restrições de idioma. A estratégia de busca incorporou os seguintes termos: “*peritoneal dialysis*”, “valsartan”, “sacubitril”, “Entresto”, “ARNI”, “*neprilysin inhibitor*” e “LCZ696”. As estratégias de busca completas para cada base de dados estão detalhadas no Apêndice Suplementar. Além disso, as listas de referências de todos os artigos incluídos foram revisadas manualmente para identificar estudos potencialmente elegíveis não capturados pelas buscas nas bases de dados.

A extração de dados foi realizada de forma independente por dois revisores (C.L. e P.E.), utilizando critérios de elegibilidade preestabelecidos e protocolos de avaliação da qualidade. As discrepâncias foram resolvidas por consenso, com o envolvimento de um terceiro autor (M.G.). Esta revisão sistemática foi registrada prospectivamente no *International Prospective Register of Systematic Reviews* (PROSPERO), sob o número de registro CRD42025643186, de acordo com as práticas de relato recomendadas.

### Desfechos

Os desfechos primários de eficácia foram as alterações absolutas em relação ao valor basal: (1) na fração de ejeção do ventrículo esquerdo (FEVE) e (2) nos níveis de NT-proBNP. Os desfechos secundários de eficácia incluíram alterações: (1) na pressão arterial sistólica (PAS), (2) no diâmetro do átrio esquerdo (DAE) e (3) na dimensão diastólica final do ventrículo esquerdo (DDVE). Além disso, os desfechos de segurança predefinidos foram a incidência de (1) hipercalemia, (2) hipotensão e (3) angioedema durante o acompanhamento.

### Avaliação de Qlualidade

O risco de viés foi avaliado por meio de ferramentas apropriadas, de acordo com o delineamento do estudo. Para os ECRs, aplicamos a ferramenta *Risk of Bias 2.0* (RoB 2) da *Cochrane Collaboration*; para estudos observacionais com grupo controle, utilizamos a *Risk of Bias in Non-randomized Studies of Interventions* (ROBINS-I); e, para estudos de braço único, foi empregado o *Joanna Briggs Institute (JBI) Critical Appraisal Checklist for Case Series* ^21,22,23^. A ferramenta RoB 2 avalia cinco domínios, classificando os estudos como apresentando “baixo risco”, “algumas preocupações” ou “alto risco” de viés. Já a ferramenta ROBINS-I avalia sete domínios, atribuindo classificações de risco de viés “baixo”, “moderado”, “sério” ou “crítico”, ou indicando “ausência de informação”. A ferramenta JBI compreende 10 itens com respostas binárias (“sim” ou “não”), nas quais pontuações cumulativas mais altas refletem um menor risco global de viés. Dois autores (C.L. e P.E.) realizaram as avaliações de forma independente, e quaisquer divergências foram resolvidas por meio de discussão com um terceiro autor.

Dada a pequena quantidade de estudos incluídos, uma avaliação formal do viés de publicação não foi viável. Gráficos de funil (*funnel plots*) não são confiáveis para detectar assimetria em meta-análises com menos de 10 estudos, e o teste de Egger possui, de forma semelhante, baixo poder estatístico em tais cenários^24^.

### Análise Estatística

Esta revisão sistemática e meta-análise foi realizada de acordo com as orientações metodológicas descritas no *Cochrane Handbook for Systematic Reviews of Interventions* e aderiu às diretrizes do *Preferred Reporting Items for Systematic Reviews and Meta-Analyses* (PRISMA)^25,26^.

Para desfechos contínuos, calculamos a *mean difference* (MD) em relação ao valor basal, com os respectivos intervalos de confiança (CI) de 95%. A significância estatística foi definida como um valor de p bicaudal < 0,05. As análises agrupadas para variáveis contínuas foram realizadas utilizando um modelo de efeitos aleatórios, com o estimador de *restricted maximum likelihood* (REML). Os desfechos de segurança foram resumidos descritivamente em uma tabela, relatando o número de eventos adversos, o número total de pacientes expostos, o número de estudos que relataram cada evento e a proporção correspondente. A heterogeneidade entre os estudos foi avaliada pelo teste Q de *Cochran* e quantificada pelo valor de I^2^; um valor de I^2^ superior a 50% foi considerado indicativo de heterogeneidade substancial. Foram realizadas análises de sensibilidade utilizando a abordagem *leave-one-out* para determinar a influência de estudos individuais nas estimativas globais agrupadas. Todas as análises estatísticas foram conduzidas no software R (versão 4.4.2; *R Foundation for Statistical Computing*, Viena, Áustria).

## Resultados

Como detalhado na Figura 1, um total de 193 estudos foi identificado mediante buscas sistemáticas em três bases de dados. Após a remoção de duplicatas e a triagem de relevância, 21 artigos em texto completo foram avaliados quanto à elegibilidade. Destes, nove estudos atenderam aos critérios de inclusão e foram incluídos na análise final, compreendendo um total de 343 pacientes com IC submetidos à DP enquanto recebiam terapia com sacubitril/valsartana^27,28,29,30,31,32,33,34,35^.

**Figura 1:** Fluxograma PRISMA de triagem e seleção dos estudos.

As características dos pacientes estão detalhadas na Tabela 1. Os estudos incluídos empregaram a diálise peritoneal ambulatorial contínua (DPAC) ou a diálise peritoneal automatizada (DPA) como modalidade de diálise. O sacubitril/valsartana foi prescrito em doses variadas entre os estudos, refletindo a prática clínica do mundo real. Os períodos de acompanhamento variaram de três a doze meses, e a média de idade dos participantes variou de 45,3 a 71 anos.

**Tabela 1:** CaracterÍsticas dos pacientes dos estudos

Estudo	Delineamento do estudo	Total de pacientes em DP	Grupo SV	Homens, n (%)	Média de idade (anos)	Modali-dade de DP	Dose de SV	Tempo de seguimento
Ding 2023	Coorte restro-spectiva	64	32	NA	NA	DPAC ou DPA	50–100 mg, BID	Mediana de 349 dias
Fu 2021	Estudo retrospectivo de braço único	21	21	14 (66,6)	51	DPAC	50–100 mg, BID	3 a 12 meses
He 2023	Estudo transversal	40	40	18 (45)	45,3	DPAC ou DPA	3 grupos: 50 mg, BID; 100 mg, QD; ou 100 mg – conforme a PA	4 meses
Ma 2023	Coorte retro-spectiva	99	61	44 (72,1)	52	DPAC ou DPA	50–100 mg, BID	6 a 12 meses
Niu 2022	Estudo caso-controle	16	10	NA	NA	NA	De 24/26 mg, BID, até 97/103 mg, BID	12 meses
Pimenta 2023	Estudo de coorte transversal	5	5	5 (100)	71	DPAC	24/26 mg, BID	Mediana de 16 meses
Sheng 2023	ECR	160	80	64 (40)	57,6	DPAC	50–100 mg, BID	6 meses
Wang 2024	Coorte retro-spectiva	102	47	63 (61,7)	56,8	DPAC	NA	12 meses
Zhang 2022	Estudo retrospectivo de braço único	47	47	28 (59,5)	45,9	DPAC ou DPA	100 mg, BID	7 dias

Abreviaturas – DP: diálise peritoneal; SV: sacubitril-valsartana; NA: não disponível; DPAC: diálise peritoneal ambulatorial contínua; DPA: diálise peritoneal automatizada; BID: duas vezes ao dia; QD: uma vez ao dia.

O tratamento com sacubitril/valsartana foi associado a uma melhora estatisticamente significativa na FEVE, com uma MD agrupada de 5,22% (95% CI, 3,86 a 6,58; p < 0,0001; I^2^ = 38,9%; Figura 2). Uma redução significativa nos níveis de NT-proBNP também foi observada (MD –5630,40 pg/mL; 95% CI, –9177,57 a –2083,23; p = 0,0019; I^2^ = 86%; Figura 3). Além disso, a PAS diminuiu significativamente após o tratamento (MD –14,59 mmHg; 95% CI, –20,59 a –8,59; p < 0,0001; I^2^ = 93,5%; Figura 4). Em contraste, as alterações no DAE e na DDVE não atingiram significância estatística. A MD agrupada para o DAE foi de –3,31 mm (95% CI, –6,70 a 0,09; p = 0,0561; I^2^ = 91,4%; Figura S1) e para a DDVE foi de –4,46 mm (95% CI, –9,83 a 0,91; p = 0,1037; I^2^ = 94,7%; Figura S2), indicando heterogeneidade substancial e efeitos inconclusivos no remodelamento estrutural.

**Figura 2:** Alterações na FEVE em pacientes em DP antes e após o tratamento com sacubitril/valsartana.

Abreviações – CI, intervalo de confiança; LVEF, fração de ejeção do ventrículo esquerdo; MD, *mean difference*; DP, diálise peritoneal; REML, *restricted maximum likelihood*; DP, desvio padrão; SV, sacubitril/valsartana.

**Figura 3:** Alterações nos níveis de NT-proBNP em pacientes em DP antes e após o tratamento com sacubitril/valsartana.

Abreviações – CI, intervalo de confiança; MD, *mean difference*; NT-proBNP, peptídeo natriurético tipo B N-terminal; DP, diálise peritoneal; REML, *restricted maximum likelihood*; DP, desvio padrão; SV, sacubitril/valsartana.

**Figura 4:** Alterações na PAS em pacientes em DP antes e após o tratamento com sacubitril/valsartana.

Abreviações – CI, intervalo de confiança; MD, *mean difference*; DP, diálise peritoneal; REML, *restricted maximum likelihood*; PAS, pressão arterial sistólica; DP, desvio padrão; SV, sacubitril/valsartana.

Em relação aos desfechos de segurança, uma incidência modesta de hipercalemia foi observada após o início do sacubitril/valsartana, com 16 eventos entre 134 pacientes (11,9%; Tabela 2). A hipotensão ocorreu com pouca frequência, com apenas 3 eventos relatados entre 206 pacientes (1,5%). Notavelmente, nenhum caso de angioedema foi identificado nos seis estudos que relataram esse desfecho, abrangendo um total de 211 pacientes em DP.

**Tabela 2:** Efeitos adversos relacionados ao uso de sacubitril/valsartana

Desfechos	Números de estudos reportando	Total de eventos	Total de pacientes	Proporção (%)
Hipotensão	6	3	206	3/206 (1,45%)
Hipercalemia	4	16	134	16/134 (11,94%)
Angioedema	6	0	211	0/211 (0%)

As análises de sensibilidade *leave-one-out* foram realizadas, excluindo-se sequencialmente cada estudo das estimativas agrupadas. Os desfechos primários de eficácia — MD em relação ao valor basal na FEVE, níveis de NT-proBNP e PAS — permaneceram estatisticamente significativos em todas as iterações, sustentando a robustez desses achados. Em contraste, os resultados agrupados para DDVE e DAE exibiram maior variabilidade após a remoção de estudos individuais, indicando estabilidade limitada e potencial sensibilidade às características de cada estudo. Cada uma dessas análises individuais pode ser encontrada no Material Suplementar. A análise de sensibilidade também destacou que certos estudos contribuíram substancialmente para a alta heterogeneidade observada em diversos desfechos.

A avaliação individual de cada estudo incluído na meta-análise é apresentada no Material Suplementar. O único ECR apresentou risco de viés classificado como “algumas preocupações”. Os estudos observacionais foram classificados como risco de viés “sério” ou “moderado”, com a ferramenta ROBINS-I, e como risco “baixo” ou “moderado”, com a ferramenta de avaliação JBI.

## Discussão

Nesta revisão sistemática e meta-análise de nove estudos, avaliamos a eficácia e a segurança do sacubitril/valsartana em uma coorte de 343 pacientes com IC submetidos à DP. Os principais achados foram os seguintes: (1) o sacubitril/valsartana foi associado a uma melhora significativa na FEVE (MD 5,22%; p < 0,0001) e a uma redução substancial nos níveis séricos de NT-proBNP (MD –5630,40 pg/mL; p = 0,0019); (2) observou-se uma diminuição significativa na PAS após o tratamento (MD –14,59 mmHg; p < 0,0001); e (3) não foram observadas alterações estatisticamente significativas no DAE ou na DDVE. Em relação aos desfechos de segurança, os eventos de hipotensão e angioedema foram raros, enquanto uma incidência modesta de hipercalemia (11,94%) foi relatada entre os pacientes que receberam sacubitril/valsartana.

O ensaio PARADIGM-HF representou um marco no desenvolvimento da terapia com INRA. Este estudo inicial demonstrou que o sacubitril/valsartana reduziu significativamente o risco de mortalidade cardiovascular e de hospitalização por IC, ao mesmo tempo em que melhorou os sintomas e as limitações físicas em pacientes com ICFER, quando comparado ao enalapril^17^. Ensaios subsequentes reforçaram ainda mais a superioridade do sacubitril/valsartana sobre os inibidores da enzima conversora de angiotensina (IECA) e os bloqueadores dos receptores de angiotensina II (BRA), particularmente na redução dos níveis de NT-proBNP e da massa ventricular esquerda^18,36,37^. Contudo, uma limitação crítica desses ensaios é a exclusão sistemática de pacientes com DRT, resultando em uma lacuna persistente na base de evidências quanto à segurança e à eficácia da terapia com INRA nesta população de alto risco.

Recentemente, duas meta-análises envolvendo pacientes com DRT em diálise foram conduzidas para preencher essa lacuna de evidências. Esses estudos demonstraram que o sacubitril/valsartana foi associado a melhoras na função e na estrutura ventricular esquerda, bem como a uma redução na mortalidade por todas as causas, sem um aumento significativo de eventos adversos^38,39^. No entanto, ambas as análises combinaram dados de pacientes em HD e DP, sendo que a maioria dos participantes realizava HD. Esta é uma limitação crítica, uma vez que o manejo da DCV em pacientes em DP difere substancialmente do manejo daqueles que se encontram em HD, devido a variações nas estratégias de remoção de fluidos, na composição do dialisato e na função renal residual. Na DP, as soluções à base de glicose, a ultrafiltração contínua, a homeostase do potássio e a preservação do estado volêmico são componentes fundamentais no manejo cardiovascular^3^. Para superar essas limitações, nossa meta-análise focou exclusivamente em pacientes submetidos à DP, permitindo uma avaliação mais direcionada do sacubitril/valsartana nesta população específica e gerando conclusões mais precisas e clinicamente relevantes quanto à sua eficácia e segurança.

Em pacientes submetidos à diálise, o remodelamento deletério da estrutura e função do ventrículo esquerdo (VE) está fortemente associado ao aumento dos riscos de morbidade e mortalidade cardiovascular^5^. Um dos principais objetivos terapêuticos da terapia com INRA é desacelerar esse remodelamento adverso, reduzindo os volumes cardíacos e melhorando a função do VE^40^. Uma meta-análise recente envolvendo mais de 10.000 pacientes demonstrou que os INRAs melhoraram os índices ecocardiográficos — incluindo a FEVE, os volumes do VE e medidas de hipertrofia^41^. Em consonância com esses achados, nossa meta-análise observou uma melhora significativa na FEVE em relação ao valor basal em pacientes em DP tratados com sacubitril/valsartana. No entanto, não foram detectadas alterações estatisticamente significativas em outros parâmetros ecocardiográficos estruturais, como o DAE e a DDVE. Uma explicação potencial para essa divergência reside na heterogeneidade das populações dos estudos. Entre os estudos que relataram o DAE e a DDVE, três em cada quatro incluíram pacientes com uma ampla gama de fenótipos de FEVE. Essa heterogeneidade clínica, que reflete variados mecanismos fisiopatológicos e de responsividade terapêutica, pode contribuir para os efeitos atenuados ou inconsistentes do sacubitril/valsartana nos parâmetros cardíacos estruturais além da FEVE^42^. Adicionalmente, o remodelamento estrutural pode vir a ser observado em um período de acompanhamento mais longo do que o dos estudos individuais incluídos nestas análises, em virtude de fatores de confusão, como a redução da sobrecarga volêmica e o melhor controle da pressão arterial com a DP^43^.

Entre pacientes em DP, níveis elevados de NT-proBNP têm sido associados ao remodelamento cardíaco adverso, incluindo hipertrofia ventricular esquerda e doença arterial coronariana^40,44^. O sacubitril/valsartana, por meio de suas propriedades de remodelamento reverso, demonstrou reduzir as concentrações circulantes de peptídeos natriuréticos, contribuindo para a melhora dos desfechos clínicos^40^. Em nossa meta-análise, observamos uma redução significativa no NT-proBNP após o tratamento, reforçando o papel do fármaco na melhora da estrutura e da função cardíaca. Esses achados sugerem uma utilidade potencial no monitoramento da progressão da doença em pacientes em DP. No entanto, devido à influência da reduzida depuração renal no acúmulo do peptídeo, sua confiabilidade na avaliação diagnóstica e prognóstica da IC em populações em diálise permanece limitada^5,39,44^. Consequentemente, as diretrizes atuais não recomendam o uso rotineiro do NT-proBNP para a avaliação da IC em pacientes recebendo diálise.

Alguns eventos adversos associados ao uso de INRA em pacientes com disfunção renal podem estar ligados a alterações farmacocinéticas. Após a administração, o sacubitril é convertido em seu metabólito ativo, o sacubitrilato, sendo eliminado primordialmente pelos rins, enquanto a valsartana é eliminada por via biliar. Dessa forma, espera-se que o comprometimento renal influencie a farmacocinética do sacubitrilato, mas não a da valsartana^45,46^. Um estudo recente avaliou os níveis de sacubitrilato em pacientes em DP e encontrou uma excreção urinária reduzida em comparação a indivíduos saudáveis. A DP pareceu ter apenas uma capacidade limitada para remover o sacubitrilato da circulação, provavelmente devido à alta ligação do fármaco às proteínas plasmáticas. Apesar dessas alterações farmacocinéticas, a exposição global ao sacubitrilato em pacientes em DP permaneceu dentro da faixa terapêutica observada na população geral, sugerindo que um esquema posológico de 100 mg duas vezes ao dia é provavelmente seguro e não requer ajuste. Esse perfil de segurança foi adicionalmente corroborado pela ausência de eventos adversos significativos nos 40 pacientes incluídos no estudo^29^.

A principal força desta revisão sistemática reside em seu foco exclusivo em pacientes em DP, uma população normalmente sub-representada em ensaios clínicos que avaliam o uso de sacubitril/valsartana. Além disso, a consistência dos resultados em todas as análises de sensibilidade reforça a robustez dos nossos achados. No entanto, nosso estudo apresenta diversas limitações. Primeiro, oito dos nove estudos incluídos eram de natureza observacional, o que pode introduzir vieses inerentes e limitar a inferência causal. Segundo, houve uma variabilidade substancial na FEVE, nas características basais da população, nos esquemas posológicos de sacubitril/valsartana e na duração do acompanhamento entre os estudos. Essas diferenças contribuíram para a heterogeneidade de moderada a alta observada em muitos dos desfechos agrupados. Embora as análises de sensibilidade não tenham revelado influência significativa de estudos individuais, os resultados devem, no entanto, ser interpretados com cautela. Terceiro, desfechos clínicos críticos — como mortalidade cardiovascular, taxas de hospitalização e mortalidade por todas as causas — não puderam ser avaliados devido ao relato limitado. Por fim, a maioria dos estudos (8 de 9) foi conduzida em países asiáticos, o que pode restringir a generalizabilidade de nossos achados para populações de pacientes mais amplas e diversificadas. Apesar dessas limitações, nosso estudo é a primeira meta-análise a avaliar o benefício do sacubitril/valsartana em pacientes em DP. Considerando que pacientes com insuficiência renal têm um risco maior de desenvolver IC, um tratamento otimizado para aqueles corretamente diagnosticados é imprescindível para melhorar a sobrevida e a qualidade de vida dos pacientes.

## Conclusão

Em conclusão, esta revisão sistemática e meta-análise sugere que o sacubitril/valsartana pode, possivelmente, oferecer benefícios clínicos para pacientes em DP. A evidência agrupada indica melhorias na FEVE, reduções nos níveis de NT-proBNP e um controle aprimorado da pressão arterial, sem um aumento considerável de eventos adversos. No entanto, a predominância de dados observacionais e a variação significativa entre os estudos impõem limitações importantes e exigem uma interpretação atenta. Apesar dessas restrições, o sacubitril/valsartana mostra-se uma opção terapêutica promissora nesta população de alto risco. Novos ECRs de alta qualidade são necessários para validar sua eficácia e segurança em pacientes submetidos à DP.

## Disponibilidade de Dados

Os conjuntos de dados gerados e/ou analisados durante o presente estudo estão disponíveis mediante solicitação razoável ao autor correspondente e encontram-se nas tabelas e figuras do manuscrito.

## Material Suplementar

O seguinte material online está disponível para o presente artigo:

Material suplementar - Estratégia de busca.
